# Face yourself! - learning progress and shame in different approaches of video feedback: a comparative study

**DOI:** 10.1186/s12909-019-1519-9

**Published:** 2019-03-27

**Authors:** Anne Herrmann-Werner, Teresa Loda, Rebecca Erschens, Priska Schneider, Florian Junne, Conor Gilligan, Martin Teufel, Stephan Zipfel, Katharina E. Keifenheim

**Affiliations:** 1 Department of Psychosomatic medicine and Psychotherapy, University Hospital Tuebingen, Osianderstr. 5, D-72076 Tuebingen, Germany; 2 Department of Child and Adolescent Psychiatry, Psychosomatics and Psychotherapy, University Hospital Tuebingen, Osianderstr. 14-16, 72076 Tuebingen, Germany; 3 School of Medicine and Public Health, faculty of Health and Medicine, University of Newcastle, Callaghan, NSW 2308 Australia; 4 Dep. of Psychosomatic Medicine and Psychotherapy, LVR Hospital Essen, University of Duisburg-Essen, Virchowstr. 14, 45147 Essen, Germany

**Keywords:** Medical students, Video feedback, Shame, Communication skills

## Abstract

**Background:**

Feedback is a crucial part of medical education and with on-going digitalisation, video feedback has been increasingly in use. Potentially shameful physician-patient-interactions might particularly benefit from it, allowing a meta-perspective view of ones own performance from a distance. We thus wanted to explore different approaches on how to deliver specifically video feedback by investigating the following hypotheses: 1. Is the physical presence of a person delivering the feedback more desired, and associated with improved learning outcomes compared to using a checklist? 2. Are different approaches of video feedback associated with different levels of shame in students with a simple checklist likely to be perceived as least and receiving feedback in front of a group of fellow students being perceived as most embarrassing?

**Methods:**

Second-year medical students had to manage a consultation with a simulated patient. Students received structured video feedback according to one randomly assigned approach: checklist (CL), group (G), student tutor (ST), or teacher (T). Shame (ESS, TOSCA, subjective rating) and effectiveness (subjective ratings, remembered feedback points) were measured. T-tests for dependent samples and ANOVAs were used for statistical analysis.

**Results:**

*n* = 64 students could be included. Video feedback was in hindsight rated significantly less shameful than before. Subjectively, there was no significant difference between the four approaches regarding effectiveness or the potential to arise shame. Objective learning success showed CL to be significantly less effective than the other approaches; additionally, T showed a trend towards being more effective than G or ST.

**Conclusions:**

There was no superior approach as such. But CL could be shown to be less effective than G, ST and T. Feelings of shame were higher before watching one’s video feedback than in hindsight. There was no significant difference regarding the different approaches. It does not seem to make any differences as to who is delivering the video feedback as long as it is a real person. This opens possibilities to adapt curricula to local standards, preferences, and resource limitations. Further studies should investigate, whether the present results can be reproduced when also assessing external evaluation and long-term effects.

**Electronic supplementary material:**

The online version of this article (10.1186/s12909-019-1519-9) contains supplementary material, which is available to authorized users.

## Background

In medical education as well as later everyday professional life, medical students are facing difficult physician-patient encounters for which they need to be trained appropriately. A crucial part of such teaching is feedback, which students receive in order to “mull over” what happened in the encounter and improve their skills [1–7]. With progressing digitalisation, video review is emerging for performance-based feedback and has been found to be particularly helpful for improving communication skills [5, 8, 9]. Studies have shown that video review is far more effective than oral feedback by teacher or peer alone [10].

Unique to the video feedback method is the ability for learners to view themselves from a meta-perspective which enables them to evaluate their own learning progress and clinical skills “from a distance” [11–14]. Fukkink and colleagues showed that through the use of video feedback, participants could improve verbal, non-verbal and paralingual aspects of their communication in a professional context [15] – i.e. key interaction skills in the physician-patient encounter.

Despite evidence for its effectiveness, facing oneself in an already apprehensive and thus potentially stressful situation can be quite embarrassing for the targeted student [10, 16–19]. This level of embarrassment might be increased when the communication situation itself has potential for awkward moments or interactions as - amongst other situations – shown for asking about psychosocial aspects or taking a sexual history [20, 21]. Embarrassment or shame – as opposed to guilt, which focusses on wrong behaviour – is essentially a negative evaluation of the complete self without any distinction between the person and its behaviour [14, 22]. The feeling of shame is known to reduce motivation and lead to subsequent avoidance of similar situations, potentially hindering the learning process [14, 19, 23]. Regarding negative emotions towards video feedback in general, Paul and colleagues [17] reported that in their study most students scored high on anxiety and resistance to videotaping beforehand, but both measures decreased after exposure to actual video feedback and with increasing practice. These findings have since been replicated, suggesting that students should be confronted with video feedback from early stages of their education on [11, 15, 24]. The same principles apply to self-evaluation and feedback techniques in general [25]. In their commentary, William and Bynum (2015) even address the issue stating that the role of shame in feedback has so far gone unrecognised in research and that there is a lack of understanding about how to effectively communicate feedback and ensure that it is received in a constructive manner [26].

At the University of Tuebingen, students are confronted from the very beginning with videotaped simulated physician-patient encounters thereby learning approaches like reflection, self-evaluation, peer-assessment, and peer-feedback as guided by models such as the Calgary-Cambridge Referenced Observation Guides and CanMeds (roles “Professional” and “Scholar”) [8, 25, 27–29]. Within the variety of models on how to implement feedback into the medical curricula, different perspectives regarding who is best to deliver feedback to the students are offered. Quite often, an instructor or teacher is responsible for giving the feedback, for example in the form of formal debriefing after simulations or within communication classes [15, 30]. However, with sometimes sparse resources, other ways are increasingly common; using student tutors for peer-assisted learning, or integrating feedback with a whole group of students [24, 31]. Some results suggest that students should best watch their videos alone and not in small groups [24]. In contrast, other authors favour group videotape review to support reconsidering one’s own approach, getting to know different techniques and encouraging each other [31]. Sharp et al. reported that students preferred feedback from a simulated patient over a private review of the video [32]. However, so far there is – to the best of our knowledge - no evidence about how to best implement video-feedback and, particularly, who is best placed to deliver it. Thus, the present study focussed on different video feedback approaches and their potential to arise shame as well as secure learning success.

## Methods

### Aim, design and setting of the study

In the present comparative study at the Medical Faculty of University of Tuebingen different video feedback approaches were investigated with regards to feeling of shame and potential learning success. We hypothesized the following:

1. The physical presence of a person delivering the feedback is more desired, and associated with improved learning outcomes compared to using a checklist.

2. Different approaches of video feedback will be associated with different levels of shame in students with a simple checklist likely to be perceived as least and receiving feedback in front of a group of fellow students being perceived as most embarrassing.

### Intervention

The intervention was implemented in an existing six module interview skills course taking place in the third pre-clinical semester. Teaching modules 3 and 4 were adapted to cover the topics “assessment of psychosocial aspects” and “taking a sexual history”. The course was held in small groups of ten students each. In the beginning of each module, the teacher introduced the theoretical background of each topic (psychosocial aspects or sexual history taking) using standardized slides and clearly defined learning goals for the session. Then, one student took on the role of the physician and managed the patient encounter with a simulated patient (SP) in front of the group. The interview was videotaped. After the interview, the student watched the videotape for feedback either independently with a checklist of expected behaviours (CL), with the whole group (G), with the teacher (T) or with a student tutor (ST). After all study-related measurements had been taken, simulated patients additionally provided their feedback as usually done in our communication classes. Assignment to one of the approaches was randomized. The randomization process was based on a random-number-approach [33], with each appointment being randomly assigned to one of the four approaches (CL, G, ST or T). Student tutors and teachers were trained with regards to suggested content of feedback as well as the feedback process in a simulated training session using a manual specifically developed for the study. Student tutors were medical students in their advanced medical training and had all gone through the course they were now tutoring as participants at earlier stages of their training. All teachers were experienced clinicians (medical doctors or clinical psychologists) regularly involved in teaching communication classes.

### Sample size

Based on experience with previous comparable interventions, sample size calculation with G*Power demanded a minimum of *n* = 12 students per group (power 0.8, significance level 0.05, effect size 0.5).

### Feedback

Independent of the feedback approach (CL, G, ST or T), the feedback process followed the same pattern. Teachers, student tutors and the group had a checklist for their feedback that corresponded with the one used in the independent CL approach (see Additional files 1 and 2). It comprised two areas with three sub-topics each. The area “conversational techniques” (general feedback points) was uniform for psychosocial aspects (PA) and sexual history (SH) with its points “introduction”, “verbal communication”, and “non-verbal communication”. The second area, was topic specific with issues concerning “profession”, “family”, and “well-being” for PA, and “sexual life quality”, “partnership quality”, and “sexual dysfunctions” for SH. On the checklist, it was evaluated whether the student had addressed each point (binary list yes/no) and if so, with what strengths/weaknesses (free comment) to guarantee standardisations as well as specific focusing which has been shown to be beneficial for beginners [34]. In the CL group, the students had to fill in the checklist independently as they worked through the video. In the G approach, feedback points were divided upon the students who had watched the encounter so that each one gave feedback to only one point, whereas the teacher and student tutor gave feedback to all 6 topics in their respective approaches (ST, T). In the end, all students acting as physicians thus received feedback on all 6 checklist areas no matter through which source (CL, G, ST or T). The video could be stopped and rewound if needed, and also student tutors and teachers were actively encouraged to do so in order to highlight difficult or successful video sequences. In the G approach, the teacher was present in the room, moderating the discussion and following instructions if fellow students wanted a certain video section to be repeated.

### Assessment

**Fig. 1:**
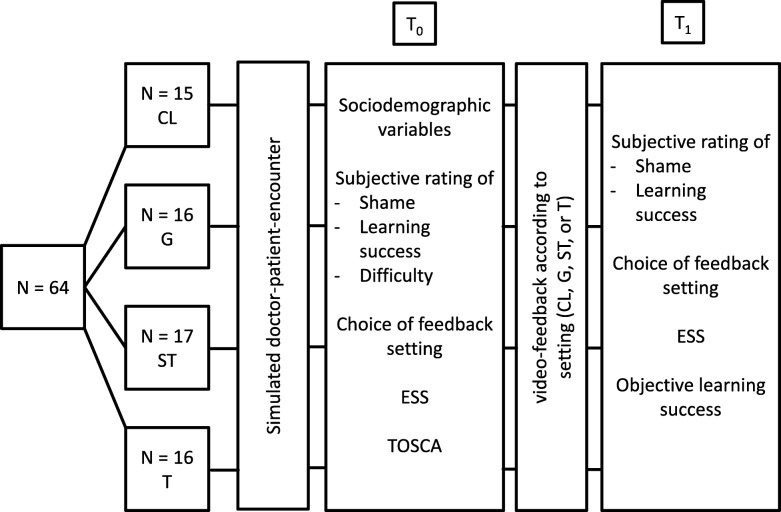
Study design with participants included, T_0_ = before receiving video feedback, T_1_ = after receiving video feedback, CL = checklist, G = group, ST = student tutor, T = teacher. ESS = Experiential Shame Scale, TOSCA = Test of Self-Conscious Affect

After having conducted the interview, but before the videotape review (T_0_), students were asked to fill in a questionnaire. It consisted of items regarding preferences about the person to deliver feedback to them (teachers, fellow students, etc.), expected learning success, shame about the teaching’s content, and video feedback in general. Additionally at T_0_, two standardized questionnaires for shame (Experiential Shame Scale (ESS) and the Test of Self-Conscious Affect (TOSCA-3)) were administered [35–37]. After having watched the videotape and having received feedback in one of the four possible feedback approaches (T_1_), students completed the same questionnaire but without TOSCA-3, and with additional general questions about shamefulness and their experience of their allocated feedback approach. Figure 1 shows an overview on the study design.

### Shame

Shame was – as described above – assessed with TOSCA-3 and ESS. The TOSCA-3 measures proneness to shame with 16 items on a 5-point Likert scale (1 = “not likely” to 5 = “very likely”; possible scores 0–80) [38]. For each scenario, a shame reaction and a guilt reaction are presented. The TOSCA-3 showed reliability and validity with Cronbach’s alpha ranging from 0.77–0.88 for the shame-proneness and from 0.70–0.83 for the guilt-proneness scale [39]. The ESS measure the actual emotion of shame in physical, emotional and social aspects of a momentary shame-reaction [40]. The ESS has been shown to reliably measure state shame with 10 items on a 7-point Likert scale and demonstrated a satisfactory internal consistency of Cronbach’s alpha ranging from 0.74–0.81 [40–42]. Besides these standardised questionnaires, students also answered questions regarding shamefulness to watch themselves in a video in general as well as in the particular feedback approach they had been randomised to, and regarding the content (PA/SH) on an 11-point Likert scale each (0 = “not at all” to 10 = “extremely”).

### Learning success

Learning success was measured subjectively through self-assessment on an 11-point Likert scale from 0 = “not at all” to 10 = “decidedly high”. To also gain a more objective measure of the effectiveness of the feedback process, it was assessed in absolute numbers (binary decision: yes/no) how many of the 6 feedback categories given were also perceived by the feedback recipient. Thus, at T_1_, participants wrote down all feedback points they could remember (blank sheet). There was only a minimal time delay between the actual video feedback and points remembered. Remembered feedback points were converted into absolute numbers, so the students could score up to 6 points.

### Statistical analysis

Mean values, associated standard deviations, frequencies and percentages of relevant factors like age, gender and approach and items like learning success were calculated. All relevant data were normally distributed. T-tests for dependent samples were conducted to compare differences of the mean values (ESS, shame questions). ANOVAS were used to test differences in shame (TOSCA) and learning success among the medical students and settings. The level of significance was *p* < .05. Statistical analysis was performed using SPSS 24 (SPSS Incorporated, Chicago, IL).

## Results

### Participants

Sixty-six medical students (59.7% female, average age 22.67 ± 3.72 years) took part in the study. Half of them had to face a simulated patient (SP) with psychological comorbidities, i.e. assess psychosocial aspects; the other half had to take a sexual history of an SP. Statistically there were no significant differences between the two scenarios regarding the difficulty rating, so they were treated as one group for further analyses. Two sets of data had to be excluded because the information about the approach (CL, G, ST or T) was missing. Of the remaining 64, *n* = 15 students were randomized to the CL approach, *n* = 16 to G, *n* = 17 to ST, and n = 16 to T. There were no significant differences between the four groups with regards to age, gender, formal medical training (e.g. nurse, paramedic) or prior experience with training in communication.

### Feedback approach

At T_0_, students of all approaches would have preferred to be in the T group (40.3%). However, at T_1_ most had a retrospective preference for ST (+ 6.0%) or G (+ 1.5%) with T still being the most popular approach in absolute numbers (*p* <. 001).

### Shame

Subjective rating of shame showed the following results: At T_0_, there was no significant difference between the four approaches (see Table 1). Overall, students considered it more shameful to watch themselves on a videotape when asked at T_0_ as compared to T_1_ (T_0_: M = 4.54 ± 2.76; T_1_: M = 3.38 ± 2.75; *p* < .001). There was again no significant difference when comparing the four approaches at T_1_ (see Table 1).

**Table 1:** Rating of shame by several instruments

Questions	Point of Time	CL (M; SD)	G (M; SD)	ST (M; SD)	T (M; SD)	p
Subjective rating of shame	T_0_	4.36 ± 2.65	3.87 ± 2.48	4.35 ± 3.16	5.69 ± 2.72	> .05
	T_1_	3.00 ± 2.35	2.87 ± 2.42	3.18 ± 2.94	4.31 ± 2.77	> .05
ESS	T_0_	35.93 ± 6.83	37.87 ± 7.73	37.59 ± 7.74	39.80 ± 7.41	> .05
	T_1_	33.38 ± 8.57	32.38 ± 7.27	33.47 ± 8.31	33.00 ± 5.85	> .05
TOSCA: shame-proneness	T_0_	44.80 ± 9.84	49.67 ± 9.64	45.06 ± 9.74	48.63 ± 6.98	> .05
TOSCA: guilt-proneness	T_0_	63.53 ± 5.28	67.27 ± 6.51	62.63 ± 7.25	64.31 ± 5.95	> .05

**Fig. 2:**
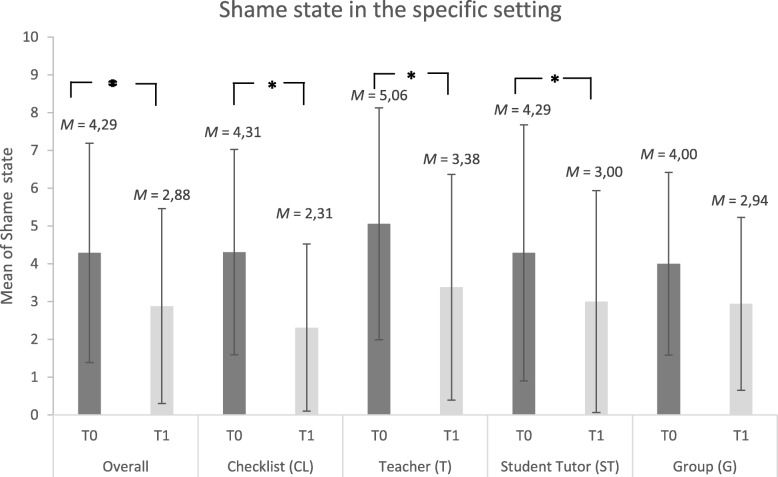
Shame to watch oneself on video in specific feedback setting (CL, G, ST, T) before (T_0_) and after (T_1_) receiving video-feedback. * indicates a significance level of *p* < .05

Similarly, students rated watching themselves on a videotape in the particular feedback approach they had been assigned to overall more shameful when asked at T_0_ as compared to T_1_ (T_0_: M = 4.29 ± 2.90; T_1_: M = 2.88 ± 2.58; *p* < .001). There were also significant effects in the approaches CL, ST and T (see Fig. 2). Looking at the topic of the encounter, PA or SH, there was no significant difference between PA and SH with regards to the potential to generate shame, and both topics were rated relatively low in this regard (PA: M = 1.59 ± 1.88, SH: M = 2.48 ± 2.29, *p* > .05). There were no significant differences between the four feedback approaches on TOSCA-3 or ESS (all *p* > 0.05, see Table 1) and results were well in line with previous studies [38, 43]

### Subjective learning success

Overall, students rated their learning success significantly higher after the video feedback than before (T_0_: M = 6.20 ± 1.93; T_1_: M = 7.20 ± 1.99; *p* < .01). At approach level, this stayed true for T (T_0_: M = 6.13 ± 1.82; T_1_: M = 7.31 ± 1.14; *p* < .05) and ST (T_0_: M = 6.06 ± 1.98; T_1_: M = 7.41 ± 1.97; p < .01). However, there was no significant difference when looking at the approaches CL or G.

### Objective learning success (remembered feedback points)

CL showed significantly lower results for remembered feedback points (M = 2.69 ± 1.65, p < .01). The three other approaches did not differ significantly (T: M = 4.20 ± 1.47; G: M = 3.94 ± 1.29; ST: M = 3.88 ± 1.75; scale 1–6), though there was a trend towards T as being most effective in conveying retained messages (F (3.56) = 2.556; *p* = .064). However, there were no significant correlations between shame scores and the ability to remember feedback points (feedback points x ESS before training: r = .002, *p* > .05; feedback points x ESS after training: r = −.036, p > .05; feedback points x TOSCA proneness to shame: r = .037, p > .05).

## Discussion

In summary, our first hypothesis could be supported as feedback approaches with a real person present were preferred and led to better learning outcomes. The CL approach, which does not enable students to discuss their performance with a peer, student tutor or teacher after watching the video scenario, was significantly less effective than the other approaches. This is in keeping with findings from past studies showing that students preferred feedback from a third party over a private review of their video [32]. Also, Sargeant and colleagues demonstrated that for improvement, accurate external feedback is necessary [44]. The CL approach might have scored lowest in the remembered feedback points due to the inadequacy of its external nature. Students rated the teacher approach best for learning outcome, which was reinforced by the fact that this approach also scored highest in relation to remembered feedback points. The fact that the student tutor was a close second to teacher, is not surprising given the fact that peer-assisted learning has been proven to be equally effective and at the same time highly valued by fellow students [45]. Further, the differences between the approaches with a person present did not differ significantly between each other, offering flexibility in the implementation of video feedback classes in the curriculum. Also, the above-mentioned idea of letting simulated patients deliver feedback to students [32], could be considered. Interestingly, preferences in our study were initially focused on the teacher but after the session, students were more open towards student tutors or even the group. These preferences might be crucial when considering different approaches as positive feelings have been shown to help students see the bigger picture rather than focus on specific details [6].

Our second hypothesis, however, did not prove to be true. There were no significant differences between the four approaches with regards to students’ feeling of shame, so receiving feedback in front of the whole group of fellow students was not perceived to be worse than more private settings like with the teacher or a student tutor in the room alone or even completely without a person. This offers flexibility regarding the application of different feedback approaches, with group and peer led sessions likely to have benefits in terms of resourcing over individual teacher-led feedback.

In line with the above mentioned research [11, 17, 24], students in the present study anticipated being video-taped and receiving feedback as much more shameful prior to the experience than they reported afterwards. Video feedback thus seems to be viewed as a positive teaching tool, but may require appropriate student preparation and reassurance before the event [16, 46]. Debriefing after a shameful experience can help the person involved to achieve personal growth through critical reflection [20, 47, 48]. In the present study, a kind of “debriefing” took place in all of the four approaches in some way – in the CL approach at least by going through the checklist again and thereby processing the encounter. This might explain the result that students’ perceived level of shame did not differ between the four approaches.

Finally, it has been stated in previous studies that feedback should always focus on the task rather than the person, to avoid generating shame [14]. The feedback given by any of the persons in our study (G, ST or T) was structured and task-focussed. This makes us believe that any experienced level of shame was not influenced by the feedback itself but by the general experience of being faced with oneself on a video [14]. The lack of difference could thus be due to methodological reasons, namely the fact that the ratings of shame were in general quite low so that the exposure might not have been challenging enough to show differences between the approaches. Or possibly, the exposure to video-feedback from the early stages of medical education, which is an important element of the Tuebingen curriculum, has lead to a certain habituation to the situation and thus generally reduced fear, shame and other negative emotions. Alternatively, students might not admit feelings of shame as it does not fit into their ideal of a physician.

There are several limitations to our study. Firstly, we only looked at one faculty, which might limit generalizability. Furthermore, learning success and perceived effectiveness have only been measured from the students’ perspective (subjective rating and remembered feedback points). In further studies it would be interesting to focus on objective measurement of learning in clinical examinations (OSCEs) or to match it with the perception of teachers, student tutors, and fellow students. However, studies have shown that external evaluation by members of the medical faculty also often lack consensus as different teachers were shown to be valuing very different aspects of the students’ performance [49, 50]. Further, there is evidence that when students rate overall instructions as effective, there is a correspondingly high perception of learning, as well as “actual learning” measured by course exams [51]. We are therefore confident that the students’ assessments are a valuable measure for the purpose of this exploratory study. Finally, we only measured immediate learning success after a single feedback experience, so it could be interesting to look at long-term follow-up results or how students perform when they have opportunities tore-rehearse. Also, we cannot exclude that the differences in measurement are due to individual backgrounds that lead to different interpretations of received feedback as described by Eva and colleagues [52].

## Conclusions

Despite these limitations we believe that our study shows how a model of video feedback teaching could be implemented. It does not seem to make any differences as to who is delivering the video feedback as long as it is a real person. This opens possibilities to adapt curricula to local standards, preferences, and resource limitations. Further studies in this field need to particularly look at long-term effects and possibilities of external evaluation.

## Additional files

Additional file 1: Title of data:: Feedback Checklist “Assessment of Psychosocial Aspects”. Description of data: Translated version of the original feedback checklist containing 6 main points - 3 general and 3 case-specific - as described in the methods section. (PDF 415 kb)

Additional file 2: Title of data:: Feedback Checklist “Taking a Sexual History”. Description of data: Translated version of the original feedback checklist containing 6 main points - 3 general and 3 case-specific - as described in the methods section. (PDF 415 kb)

## Abbreviations

CL: Checklist
ESS: Experiential Shame Scale
G: Group
OSCE: Objective measurement of learning in clinical examinations
PA: Psychosocial aspects
SH: Sexual history
SP: Simulated patient
ST: Student tutor
T: Teacher
TOSCA: Test of Self-Conscious Affect
