# Supplementary material for: Face yourself! - learning progress and shame in different approaches of video feedback: a comparative study
Source: BMC Med Educ. 2019 Mar 27;19:88. doi: 10.1186/s12909-019-1519-9 (PMC6437998; doi:10.1186/s12909-019-1519-9)
Supplement: Supplementary file 1 — Title of data:: Feedback Checklist “Assessment of Psychosocial Aspects”. Description of data: Translated version of the original feedback checklist containing 6 main points - 3 general and 3 case-specific - as described in the methods section. (PDF 415 kb) [file 12909_2019_1519_MOESM1_ESM.pdf]

iTüpFerl („in Tübingen psychosoziale Fertigkeiten erlangen)

Feedback Checklist – Assessment of Psychosocial Aspects

Instruction: *Please cross "yes" or "no" according to whether the student you have observed addressed the respective point. Under "how?" you should explain why you crossed "yes" or "no". Please provide feedback to the student you have observed according to your points on this checklist.*

1. Specific Feedback Points

1.1 Profession:

Assessment of satisfaction and stress at work?

☐ yes ☐ no how?

Example for "how?": The assessment of the patient's profession and satisfaction at work was successfully made, but the assessment of stress was missing.

1.2 Family:

Assessment of conflicts/support from the family system?

☐ yes ☐ no how?

1.3 Well-being:

Assessment of subjective feelings of stress due to the illness (specifically regarding mood, sleep)?

☐ yes ☐ no how?

2. General Feedback Points

2.1 Introduction:

Reference to own name and professional role/function (e.g. student, doctor in training, medical doctor)?

☐ yes ☐ no how?

2.2 Verbal Communication:

Adequate language (e.g. no technical terminology, well-structured sentences)?

☐ yes ☐ no how?

2.3 Non-verbal Communication:

Holding eye contact (specifically in difficult situations)?

☐ yes ☐ no how?
