# Supplementary material for: Face yourself! - learning progress and shame in different approaches of video feedback: a comparative study
Source: BMC Med Educ. 2019 Mar 27;19:88. doi: 10.1186/s12909-019-1519-9 (PMC6437998; doi:10.1186/s12909-019-1519-9)
Supplement: Supplementary file 2 — Title of data:: Feedback Checklist “Taking a Sexual History”. Description of data: Translated version of the original feedback checklist containing 6 main points - 3 general and 3 case-specific - as described in the methods section. (PDF 415 kb) [file 12909_2019_1519_MOESM2_ESM.pdf]

Feedback Checklist – Taking a Sexual History

Instruction: *Please cross "yes" or "no" according to whether the student you have observed addressed the respective point. Under "how?" you should explain why you crossed "yes" or "no". Please provide feedback to the student you have observed according to your points on this checklist.*

1. Specific Feedback Points

1.1 Sexual Life Quality:
Assessment of satisfaction with sexual life?
☐ yes ☐ no how? ____________________ ____________________

Example for "how?": The assessment of the patient's sexual life in general took place, but there was no specific question regarding satisfaction.

1.2 Partnership Quality:
Assessment of satisfaction within the relationship?
☐ yes ☐ no how? ____________________ ____________________

1.3 Sexual Dysfunctions:
Assessment of sexual dysfunctions and complaints?
☐ yes ☐ no how? ____________________ ____________________

2. General Feedback Points

2.1 Introduction:
Reference to own name and professional role/function (e.g. student, doctor in training, medical doctor)?
☐ yes ☐ no how? ____________________ ____________________

2.2 Verbal Communication:
Adequate language (e.g. no technical terminology, well-structured sentences)?
☐ yes ☐ no how? ____________________ ____________________

2.3 Non-verbal Communication:
Holding eye contact (specifically in difficult situations)?
☐ yes ☐ no how? ____________________ ____________________
